# Supplementary material for: Longitudinal study of humoral immunity against SARS-CoV-2 of health professionals in Brazil: the impact of booster dose and reinfection on antibody dynamics
Source: Front Immunol. 2023 Jul 14;14:1220600. doi: 10.3389/fimmu.2023.1220600 (PMC10376701; doi:10.3389/fimmu.2023.1220600)
Supplement: Supplementary file 1 [file Image_1.pdf]

Supplementary Material

Longitudinal study of humoral immunity against SARS-CoV-2 of health professionals in Brazil: the impact of booster dose and reinfection on antibody dynamics

Ana Paula Moreira Franco-Luiz[1], Nubia Monteiro Gonçalves Soares Fernandes[1], Thais Bárbara de Souza Silva[2], Wilma Patrícia de Oliveira Santos Bernardes[1], Mateus Rodrigues Westin[3], Thais Garcia Santos[1], Gabriel da Rocha Fernandes[4], Taynãna César Simões[5], Eduardo Fernandes e Silva[6], Sandra Grossi Gava[7], Breno Magalhães Alves[8], Mariana de Carvalho Melo[8], Rosiane A. da Silva-Pereira[1], Pedro Augusto Alves[2], Cristina Toscano Fonseca[1]*

*** Correspondence:** Cristina Toscano Fonseca: cristina.toscano@fiocruz.br

1.1 Supplementary Figures

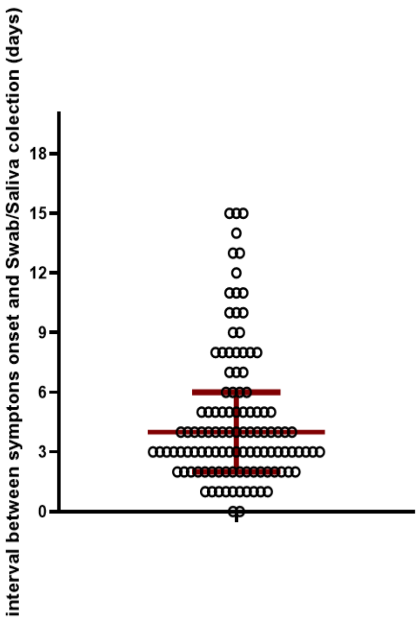

Supplementary Figure S1. Interval between symptons onset and Swab/Saliva colection (days). The points represent the interval of days between the onset of suspected symptoms and the collection of saliva or nasopharyngeal samples. The media with interquartile range are represented by red line.
